# Prognostic value of platelet distribution width to lymphocyte ratio in patients with hepatocellular carcinoma following hepatectomy

**DOI:** 10.1186/s12885-023-11621-8

**Published:** 2023-11-16

**Authors:** Zhi-Han Zhong, Lei Liang, Tian-Wei Fu, Mu-Gen Dai, Jian Cheng, Si-Yu Liu, Tai-Wei Ye, Guo-Liang Shen, Cheng-Wu Zhang, Dong-Sheng Huang, Jun-Wei Liu

**Affiliations:** 1 General Surgery, Cancer Center, Department of Hepatobiliary & Pancreatic Surgery and Minimally Invasive Surgery, Affiliated People’s Hospital, Zhejiang Provincial People’s Hospital, Hangzhou Medical College, Hangzhou, 310014 Zhejiang China; 2 Department of Gastroenterology, The Fifth Affiliated Hospital of Wenzhou Medical University, Wenzhou, Zhejiang Province China; 3 Department of Laboratory Medicine, The Key Laboratory of Imaging Diagnosis and Minimally Invasive Interventional Research of Zhejiang Province, Zhejiang University Lishui Hospital, Lishui, Zhejiang China; 4 Department of the Second School of Clinical Medicine, Zhejiang Chinese Medical University, Hangzhou, Zhejiang China

**Keywords:** Hepatocellular carcinoma, Hepatectomy, Platelet distribution width, Platelets-lymphocyte-ratio, Prognosis

## Abstract

**Background:**

Platelet distribution width (PDW), but not platelet count, was found to more comprehensively reflect platelet activity. The present study, thus, aimed to evaluate the prognostic value of PDW to lymphocyte ratio (PDWLR) in patients with hepatocellular carcinoma (HCC) following hepatectomy.

**Methods:**

Patients following hepatectomy were analyzed retrospectively. The Kaplan-Meier survival curves and Cox regression model were used to determine the prognostic value of PDWLR.

**Results:**

241 patients were analyzed eventually, and stratified into low and high PDWLR groups (≤ 9.66 vs. > 9.66). Results of comparing the baseline characteristics showed that high PDWLR was significantly associated with cirrhosis, and intraoperative blood loss (all P < 0.05). In multivariate COX regression analysis, PDWLR was demonstrated as an independent risk factor for OS (HR: 1.549, P = 0.041) and RFS (HR: 1.655, P = 0.005). Moreover, PDWLR demonstrated a superior capacity for predicting prognosis compared to other indicators.

**Conclusion:**

Preoperative PDWLR has a potential value in predicting the prognosis of HCC patients following hepatectomy, which may help in clinical decision-making for individual treatment.

## Introduction

Hepatocellular carcinoma (HCC) is the sixth most common cancer in the world, with rising morbidity [1]. So far, hepatectomy is still one of the optimum curative treatments for resectable HCC. However, the long-term prognosis of patients is still far from satisfactory, because nearly 70% of patients have recurrence or metastasis within 5 years after surgery [2, 3]. Therefore, it is very important and urgent to find effective preoperative biomarkers to identify a high-risk patient with a poor prognosis and provide personalized treatment to improve survival.

Platelets play multiple roles in cancer progression and metastasis. Activated platelets play a mediating role in tumor growth, invasion, abnormal angiogenesis, and metastasis [4, 5]. It has been proved that platelet elevation is associated with adverse outcomes of many different cancers, including gastric cancer, pancreatic cancer, ovarian cancer, and endometrial cancer [6–9]. Meanwhile, lymphocytes are the key cell component of immune response and participate in the anti-tumor immune process. It has been studied that the increase of tumor lymphocyte infiltration on the surface is related to the improvement of patients’ prognosis [10]. Therefore, previous studies have used platelet-to-lymphocyte ratio (PLR) as an inflammatory indicator and proved that PLR is a potentially valuable indicator for predicting adverse outcomes of HCC patients [11–13].

However, platelet count per se does not indicate platelet activity, but rather platelet size reflects its activity. Platelet distribution width (PDW) was found to comprehensively reflect platelet activity, which is an indicator of changes in platelet size [14]. Compared with the rapidly changing platelet count, PDW has been revealed as a better indicator to reflect the characteristics of activated platelets [15]. Recently, PDW has also been proven to be a prognostic marker of HCC, which is significantly related to the survival of HCC patients after hepatectomy [16]. However, relying solely on a single indicator such as PDW may not accurately reflect the patient’s immune response. Therefore, the purpose of this study is to investigate the potential usefulness of a new index, PDW to lymphocyte ratio (PDWLR), in predicting the prognosis of patients with HCC, to provide a more comprehensive and realistic reflection of the patient’s immune response state.

## Patients and methods

### Study population and inclusion criteria

From October 2014 to October 2019, a retrospective analysis was conducted on 241 HCC patients who underwent curative hepatectomy (R0) at Zhejiang Provincial People’s Hospital. The institutional review board at Zhejiang Provincial People’s Hospital approved this study (No.QT2022430) and all patients’ informed consent was obtained before surgery. The study followed the Strengthening the Reporting of Observational Studies in Epidemiology (STROBE) reporting guideline. The process of this study was guided by the Declaration of Helsinki. The inclusion criteria were as follows: (1) Patients with HCC were confirmed by pathology, (2) No history of other malignant tumors, (3) No extrahepatic metastasis, (4) Patients with complete medical records, (5) Age > 18 years. The exclusion criteria were as follows: (1) Recurrent HCC, (2) Patients died within 3 months after hepatectomy, (3) Patients with immune system diseases or blood system diseases, (4) Patients with acute or chronic infection within 2 weeks, (5) Patients received any anti-tumor therapy before the operation, (6) Patients with a history of splenectomy, (7) Patients receiving anticoagulation and/or antiplatelet therapy.

### Data acquisition and followed-up

The following variables were analyzed: clinical characteristics of patients: age, sex, and cirrhosis; preoperative laboratory indexes: alpha-fetoprotein (AFP), platelet (PLT) count, total bilirubin (TBIL), alanine aminotransferase (ALT), aspartate aminotransferase (AST), PT, albumin (ALB), and PDWLR; pathological features: tumor size, number, degree of differentiation, and microvascular invasion (MVI); surgical variables: intraoperative blood loss, and resection margin (< 1 cm vs. ≥ 1 cm). The preoperative laboratory indexes were collected as the latest hematological parameters one week before surgery. The calculation formula of PDWLR was: PDWLR = platelet distribution width(fl.)/lymphocyte count (10^9^/L). All patients were followed up after hospital discharge. Postoperative surveillance included physical examination, serum AFP level, ultrasonography or contrast-enhanced computed tomography (CT) or magnetic resonance imaging (MRI) of the chest and abdomen at least once every two months in the first 6 months after liver resection, and then every three months in the following 18 months, and at 6-monthly intervals thereafter. CT, MRI, angiography, bone scan, or positron emission tomography were performed earlier when recurrence or distant metastasis was suspected. Further treatment for the recurrent tumor was evaluated at each center by multidisciplinary discussion. Overall survival (OS) is calculated as the time frame from the date of operation to either the date of death or the last follow-up date. Recurrence-free survival (RFS) is calculated as the time interval between surgery and the date of disease recurrence.

### Statistical analysis

The Chi-square (χ2) test or the Fisher exact test was used to compare the classified variables. The ROC curve was used to determine the optimal cut-off value of PDWLR. The Kaplan-Meier survival curve was used to show the survival difference between the two groups and compared by log-rank test. Univariate and multivariate Cox proportional hazard regression models were performed to determine the independent prognostic factors. Time-dependent ROC was used to compare the ability of each indicator to predict prognosis. Hazard ratio (HR) and 95% confidence interval (CI) were used to describe relative risk factors. P < 0.05 was set as statistically significant. All statistical analyses were performed using SPSS version 25.0 (IBM SPSS Statistics 25.0) and the software of R 4.2.3 (http://www.r-project.org/).

## Results

### Baseline characteristics

This study eventually included a total of 241 patients. The optimal cut-off value of PDWLR as determined by the ROC curve was 9.66. Subsequently, all 241 patients were stratified into low PDWLR (≤ 9.66, n = 119) and high PDWLR (> 9.66, n = 122) groups. While there were no statistical differences observed in most of the relevant variables amongst the baseline characteristics between the two groups (all P > 0.05), patients with high PDWLR exhibited poorer liver function and higher incidences of cirrhosis (84.4% vs. 62.2%, P < 0.001), as well as a greater likelihood of experiencing intraoperative bleeding (41.0% vs. 27.7%, P < 0.001) (Table 1).

**Table 1:** Comparisons of clinical characteristics between the two groups according to preoperative PDWLR.

Variable (N, %)		PDWLR	
	≤ 9.66 (119, 49.3)	> 9.66 (122, 50.6)	P
Sex, male	107 (89.9)	105 (86.1)	0.430
Age, > 60 years	50 (42.0)	50 (41.0)	0.871
PLT, > 100 *10^9^ /L	112 (94.1)	77(63.1)	< 0.001
TBIL, > 17.1 µmol/L	38 (31.9)	61(50.0)	0.006
ALT, > 40 U/L	40 (33.6)	35 (28.7)	0.409
AST, > 40 U/L	43 (36.1)	52 (42.6)	0.303
PT, > 13.5 s	6 (5.0)	16 (13.1)	0.042
ALB, > 35 g/L	104 (87.4)	95 (77.9)	0.062
AFP, > 400 ng/mL	22 (18.5)	37 (30.3)	0.037
Cirrhosis, yes	74 (62.2)	103 (84.4)	< 0.001
Tumor size, ≤ 5/> 5 cm	81(68.1)/38(31.9)	85(69.7)/37(30.3)	0.889
Tumor number, 1/≥ 2	99(83.2)/20(16.8)	102(83.6)/20(16.4)	1.000
Satellite nodes, yes	12 (10.1)	13 (10.7)	1.000
Capsule, incomplete	79 (66.4)	88 (72.1)	0.402
Resection margin, < 1/≥ 1	38(31.9)/81(68.1)	30(24.6)/92(75.4)	0.252
MVI, yes	45(37.8)	56(45.9)	0.203
Poor differentiation	23 (19.3)	34 (27.9)	0.116
Intraoperative blood loss, > 400 mL	33(27.7)	50(41.0)	0.042
Operation time, > 180 min	42 (35.3)	39 (32.0)	0.589
Blood transfusion, yes	27 (22.7)	27 (22.1)	1.000

PDWLR, of platelet distribution width to lymphocyte ratio; PLT, platelet; TBIL, total bilirubin; ALT, alanine aminotransferase; AST, aspartate aminotransferase; PT, prothrombin time; ALB, Albumin; AFP, alpha-fetoprotein; MVI, microvascular invasion

### Survival outcomes

**Fig. 1:**
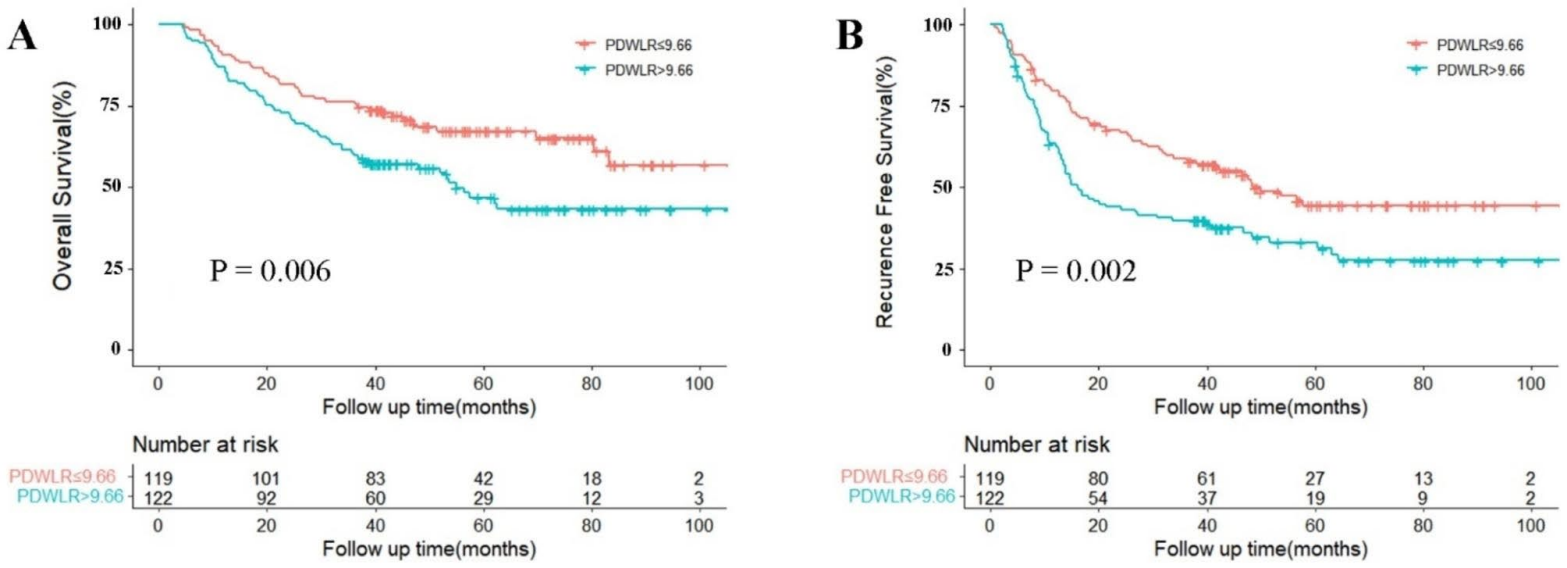
Curves comparisons of overall survival (**A**) and recurrence-free survival (**B**) between the two groups (calculated by log-rank test). PDWLR, platelet distribution width to lymphocyte ratio

After a median follow-up of 54.2 months, 140 (58%) patients experienced tumor recurrence and 101 (42%) patients died. For the entire cohort, the 1-, 3-, and 5-year OS were 87%, 67%, and 57%, while the 1-, 3-, and 5-year RFS were 67%, 46%, and 35%, respectively. The low PDWLR group showed a better 1-, 3-, and 5-year OS of 90%, 74%, and 66%, respectively, compared to the high PDWLR group which had an OS of 83%, 60%, and 48%, respectively (Fig. 1A). Similarly, the low PDWLR group had a better 1-, 3-, and 5-year RFS of 76%, 54%, and 41%, respectively, compared to the high PDWLR group which had an RFS of 58%, 39%, and 29%, respectively (Fig. 1B). The log-rank test using the Kaplan-Meier survival curve indicated that high PDWLR played a negative effect on both OS and RFS for patients with HCC after hepatectomy (P = 0.006 and P = 0.002, respectively).

### Independent-risk factors of overall survival and recurrence-free survival

Variables with a P-value < 0.1 in the univariate Cox regression analysis were entered into the forward stepwise multivariate Cox proportional hazard regression analysis, and the results are presented in Tables 2 and 3. The findings suggested that high PDWLR was an independent risk factor for both OS (HR:1.549, 95%CI 1.018–2.357, P = 0.041) and RFS (HR:1.655, 95%CI:1.160–2.360, P = 0.005). To avoid collinearity, PLR, PDW, and PLT were analyzed separately with other variables, and the results indicated that PLR was also an independent risk factor for both OS (HR: 1.144, 95%CI: 1.009–1.513, P = 0.042) and RFS (HR: 1.109, 95%CI: 1.025–1.458, P = 0.027). However, the PDW and PLT were not found to be significant independent risk factors for OS (HR: 1.005, 95%CI: 0.876–1.305, P = 0.125; HR: 1.309, 95%CI: 0.841–1.357, P = 0.307, respectively) or RFS (HR: 1.105, 95%CI: 0.894–1.346, P = 0.061; HR: 1.108, 95%CI: 0.611–1.284, P = 0.619, respectively).

**Table 2:** Univariable and multivariable Cox regression analyses of risk factors associated with overall survival for HCC after hepatectomy

Variables, comparison	UV HR (95% CI)	UV P	MV HR (95% CI)^#^	MV P
Sex, female vs. male	0.755 (0.393–1.450)	0.398		
Age, > 60 vs. ≤ 60 years	0.876 (0.588–1.305)	0.514		
TBIL, > 17.1 vs. ≤ 17.1 µmol/L	1.026 (0.690–1.527)	0.898		
ALT, > 40 vs. ≤ 40 U/L	1.285 (0.855–1.932)	0.227		
AST, > 40 vs. ≤ 40 U/L	1.278 (0.863–1.892)	0.221		
PT, > 13.5 vs. ≤ 13.5 s	1.358 (0.726–2.541)	0.338		
ALB, > 35 vs. ≤ 35 g/L	2.419 (1.586–3.732)	<0.001	1.563 (0.895–2.168)	0.056
AFP, > 400 vs. ≤ 400 µg/L	1.859 (1.226–2.819)	0.004	1.102 (1.015–1.721)	0.031
Cirrhosis, yes vs. no	1.399 (0.872–2.244)	0.164		
Tumor size, > 5 vs. ≤ 5 cm)	3.175 (2.145–4.701)	<0.001	2.192 (1.404–3.421)	<0.001
Tumor number, ≥ 2 vs. 1	1.825 (1.144–2.912)	0.012	1.249 (1.065–2.040)	0.035
Satellite nodes, yes vs. no	1.744 (1.096–3.054)	0.042	1.546 (1.061–2.643)	0.019
Incomplete capsule, yes vs. no	3.564 (2.033–6.246)	0.001	1.017 (1.004–1.729)	0.041
Tumor margin, ≥ 1 vs. <1 cm	1.821 (1.212–2.735)	0.004	1.602 (1.144–2.047)	0.020
MVI, yes vs. no	2.858 (1.912–4.273)	<0.001	1.680 (1.063–2.654)	0.026
Intraoperative blood loss > 400 vs. ≤ 400 mL	1.338 (0.881–2.031)	0.172		
Operation time ≥ 180 vs. < 180 min	1.874 (0.792–1.779)	0.405		
Blood transfusion, yes vs. no	0.852 (0.531–1.366)	0.505		
PDWLR, > 9.66 vs. ≤ 9.66	1.741 (1.167–2.596)	0.007	1.549 (1.018–2.357)	0.041
PLR*, > 128.1 vs. ≤ 128.1	1.359 (1.159–1.681)	0.033	1.144 (1.009–1.513)	0.042
PDW*,> 13.7% vs. ≤ 13.7%	1.201 (1.015–1.421)	0.029	1.005 (0.876–1.305)	0.125
PLT*, > 100 vs. ≤ 100 *10^9^ /L	1.044 (0.651–1.676)	0.857	1.309 (0.841–1.357)	0.307

^**#**^Those variables found significant at P < 0.1 in univariable analyses were entered into multivariable analyses. ^*^Those variables were analyzed separately from other variables. PDWLR, ratio of platelet distribution width to lymphocyte; PLR, platelets-lymphocyte-ratio; PDW, platelet distribution width; PLT, platelet; TBIL, total bilirubin; ALT, alanine aminotransferase; AST, aspartate aminotransferase; PT, Prothrombin time; ALB, albumin; AFP, alpha-fetoprotein; MVI, microvascular invasion; MV, multivariable; UV, univariable; HR, hazard ratio

**Table 3:** Univariable and multivariable Cox regression analyses of risk factors associated with recurrence-free survival for HCC after hepatectomy

Variables, comparison	UV HR (95% CI)	UV P	MV HR (95% CI)	MV P
Sex, female vs. male	0.666 (0.376–1.180)	0.164		
Age, > 60 vs. ≤ 60 years	0.835 (0.595–1.171)	0.295		
TBIL, > 17.1 vs. ≤ 17.1 µmol/L	1.274 (0.913–1.780)	0.155		
ALT, > 40 vs. ≤ 40 U/L	1.151 (0.860–2.571)	0.127		
AST, > 40 vs. ≤ 40 U/L	0.899 (0.574–1.407)	0.640		
PT, > 13.5 vs. ≤ 13.5 s	1.455 (0.851–2.487)	0.171		
ALB, > 35 vs. ≤ 35 g/L	1.633 (0.887–2.453)	0.208		
AFP, > 400 vs. ≤ 400 µg/L	1.670 (1.159–2.405)	0.006	1.547 (1.082–2.211)	0.017
Cirrhosis, yes vs. no	1.455 (0.981–2.160)	0.063	1.114 (1.012–2.036)	0.043
Tumor size, > 5 vs. ≤ 5 cm)	2.892 (2.066–4.049)	<0.001	2.259 (1.519–3.358)	<0.001
Tumor number, ≥ 2 vs. 1	2.323 (1.572–3.433)	<0.001	1.651 (1.091–2.497)	0.018
Satellite nodes, yes vs. no	2.391 (1.463–3.907)	0.001	1.591 (1.161–2.634)	0.031
Incomplete capsule, yes vs. no	2.285 (1.507–3.466)	<0.001	1.845 (1.194–2.850)	0.006
Tumor margin, ≥ 1 vs. <1 cm	1.541 (1.083–2.194)	0.016	1.358 (1.088–1.953)	0.029
MVI, yes vs. no	3.006 (2.136–4.228)	<0.001	2.011 (1.375–2.941)	<0.001
Intraoperative blood loss > 400 vs. ≤ 400 mL	1.211 (0.841–1.744)	0.303		
Operation time ≥ 180 vs. < 180 min	1.152 (0.795–1.668)	0.456		
Blood transfusion, yes vs. no	0.884 (0.590–1.324)	0.549	0.958 (0.637–1.441)	0.837
PDWLR, > 9.66 vs. ≤ 9.66	1.682 (1.202–2.354)	0.002	1.655 (1.160–2.360)	0.005
PLR*, > 128.1 vs. ≤ 128.1	1.476 (1.105–1.649)	0.016	1.109 (1.025–1.458)	0.027
PDW*,> 13.7% vs. ≤ 13.7%	1.209 (1.003–1.521)	0.037	1.105 (0.894–1.346)	0.061
PLT*, > 100 vs. ≤ 100 *10^9^ /L	1.007 (0.672–1.507)	0.975	1.108 (0.611–1.284)	0.619

^**#**^Those variables found significant at P < 0.1 in univariable analyses were entered into multivariable analyses. ^*^Those variables were analyzed separately from other variables. PDWLR, ratio of platelet distribution width to lymphocyte; PLR, platelets-lymphocyte-ratio; PDW, platelet distribution width; PLT, platelet; PLT, platelet; TBIL, total bilirubin; ALT, alanine aminotransferase; AST, aspartate aminotransferase; PT, prothrombin time; ALB, albumin; AFP, alpha-fetoprotein; MVI, microvascular invasion; MV, multivariable; UV, univariable; HR, hazard ratio

### Performance to predict the prognosis

**Fig. 2:**
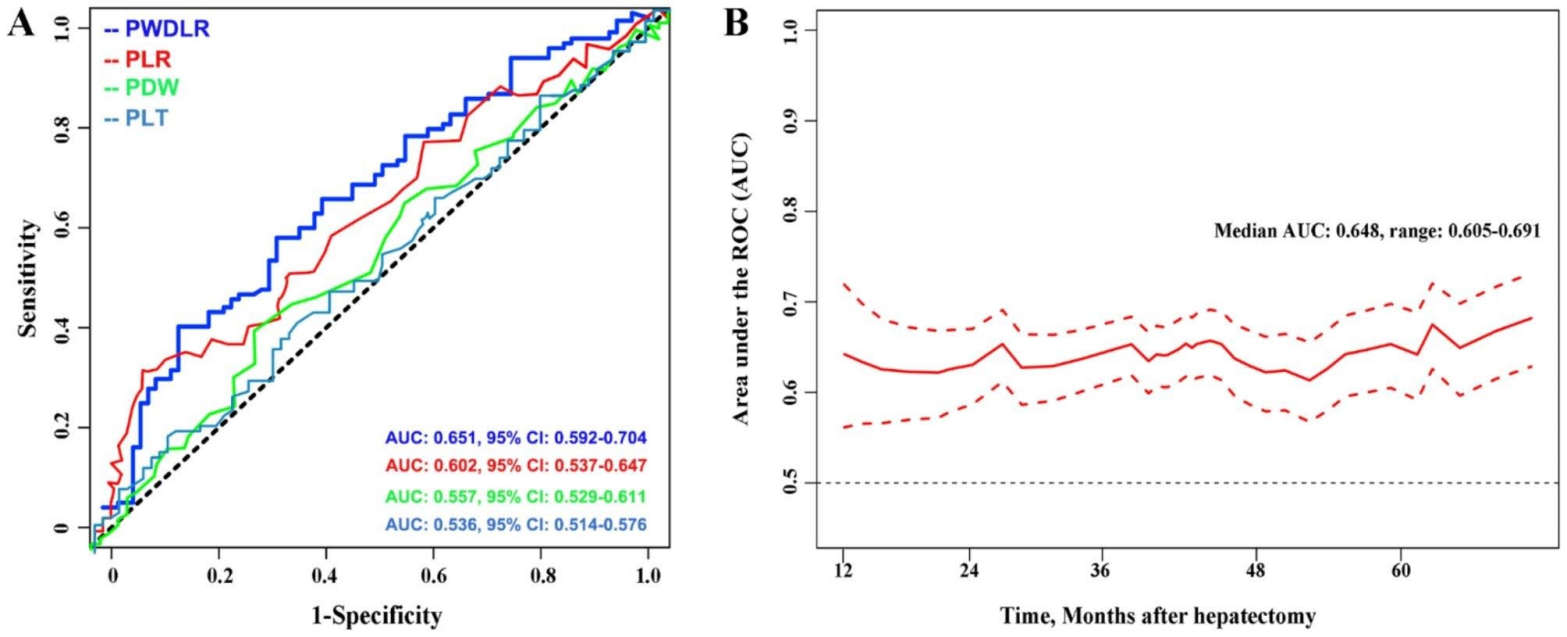
** A.** Comparison of the predictive ability of postoperative overall survival at 5-year by time-dependent ROCs between the PDWLR and the other indicators. **B.** Median AUC of PDWLR at each time. ROCs, receiver operating characteristic curves; AUCs: areas under the curves; PDWLR, platelet distribution width to lymphocyte ratio; PLR, platelets-lymphocyte-ratio; PDW, platelet distribution width; PLT, platelet

The area under the time-dependent ROC curve was calculated to determine which indicator was better at predicting survival. Initially, we evaluated the model’s ability to predict overall survival at five years, as shown in Fig. 2A. The AUC value for PDWLR was 0.651 (95% CI 0.592–0.704), indicating that it had a higher diagnostic capacity than PLR (AUC: 0.602, 95% CI 0.537–0.647), PDW (AUC: 0.557, 95% CI 0.529–0.611), and PLT (AUC: 0.536, 95% CI 0.514–0.576) (all P < 0.05). Therefore, PDWLR had the highest ability to predict overall survival compared to the other indicators at five years. Furthermore, we calculated the estimated AUC with a 95% confidence interval at different time points using time-dependent ROC curves (Fig. 2B). The results showed that the AUC of PDWLR was stable, with a median AUC of 0.648 (range 0.605–0.691).

## Discussion

In this study, we discussed the correlation between PDWLR and the prognosis of HCC patients for the first time. Using ROC analysis, the best cut-off value of PDWLR for predicting the prognosis of HCC patients was determined at 9.66. Additionally, we found that PDWLR increased with poorer liver function and higher incidences of cirrhosis, as well as a greater likelihood of experiencing intraoperative bleeding. Moreover, PDWLR was identified as an independent risk factor for predicting the prognosis of HCC patients, and it has a better and more stable ability than other indicators to predict patients’ long-term survival after surgery.

Growing interest is recently being focused on the role played by the platelets in favoring HCC growth and metastasis. Platelets are disc-shaped cells that lack nuclei [16]. Since initially discovered in 1882, our knowledge regarding the critical function of platelets in hemostasis and thrombosis has grown tremendously [17]. However, beyond their role in regulating hemostasis and coagulation, multiple pieces of evidence now suggest that platelets serve much broader functions in various diseases. Recent discoveries have revealed that platelets actively participate in numerous physiological and pathological processes, including but not limited to innate and adaptive immune responses, and carcinogenesis. Thrombocytosis, considered a paraneoplastic syndrome, may occur during the natural course of neoplastic progression, frequently accompanying cancer growth and metastatic dissemination [18–20]. High platelet count has been correlated with poor prognosis in patients with various malignancies.

Platelets were not always considered to be a single prognostic determinant, and ratios of biochemical tests, as well as formulas including platelet count, were tested as potential predictors of prognosis in patients with HCC [21]. Lymphocytes have been shown to play an important role in cancer immune monitoring and the prevention of malignant tumor development [22]. A pro-inflammatory state can lead to impaired cell-mediated immunity and the impaired response of T lymphocytes induced by cytokines [23]. The reduction of CD4^+^ T helper lymphocytes may cause a poor lymphocyte-mediated immune response to tumor cells [24]. More specifically, the PLR has been investigated as a useful prognostic tool in several types of cancer, including HCC. The PLR was consistently associated with both overall and recurrence-free survivals after HCC resection, with high values being associated with poorer outcomes.

However, platelet count per se does not indicate platelet activity, but rather platelet size reflects its activity. The variability and heterogeneity of platelet volume can be reflected by PDW. When both mature and immature platelets are present in the circulation, the value of PDW increases. This indicates that an increase in PDW can lead to a higher possibility of abnormal thrombosis [25], and also indicates differentiation of megakaryocyte heterogeneity [26]. Therefore, as the tumor grows and various proinflammatory cytokines are up-regulated, these cytokines promote the maturation of heterogeneous megakaryocytes, which allows immature platelets to enter the circulation to meet the increasing demand. At the same time, the size and characteristics of these platelets are different, ultimately increasing PDW.

Thus, we propose a new indicator, PDWLR, which provides relatively accurate information about the prognosis of HCC patients, and is non-invasive and easy to obtain in clinical practice. PDW is a platelet-related marker, which reflects the activity of platelets. This is an interactive process. Tumors can secrete factors that promote the differentiation and proliferation of megakaryocytes, and increase the production and activation of platelets. Conversely, activated platelets can prevent tumor cells from being lysed, release growth factors, and promote tumor growth and metastasis [27]. Lymphocytes can affect the survival of patients through the mechanism of inhibiting tumor survival or proliferation [28]. Therefore, high PDWLR means high platelet activity and low lymphocyte count, which indicates that the prognosis of patients with HCC is poor. This is consistent with our research results.

The present study also demonstrated that PDWLR was associated with the recurrence of HCC. First, activated platelets are more likely to cover tumor cells, and tumor cells covered by platelets will escape the attack of the autoimmune system [29]. PDW is a good indicator of platelet activation. Secondly, when the immunity decreases, especially when the microenvironment where the tumor is located is immune abnormal, resulting in the corresponding lymphocytes being unable to successfully generate an immune response, the tumor will escape the surveillance from the immune system. When the tumor has immune tolerance or immune escape, it is more likely to have further progress. Therefore, patients with higher PDWLR values are more likely to relapse. This study also shows that patients with higher PDWLR are more likely to relapse. Therefore, for patients with high PDWLR, more frequent reexamination may be required after surgery to detect tumor progression and recurrence in time.

This study also has some limitations. First of all, the number of patients included is small, and this study is a single-center study. Secondly, retrospective studies inevitably lead to data collection bias. In addition, the changes in inflammatory indicators are affected by many factors, not only tumors but also different regions and races. Therefore, the reliability of our conclusion needs further research to prove.

## Conclusions

The results of this study confirm that PDWLR is an independent predictor of RFS and OS in HCC patients. In addition, PDWLR is a potential prognostic indicator for HCC patients because of its simplicity and low cost in clinical practice.

## Data Availability

The datasets used and analyzed during the current study are available from the corresponding author upon reasonable request.

## List of abbreviations

HCC: hepatocellular carcinoma
PDW: platelet distribution width
PLR: platelets-lymphocyte-ratio
ALT: alanine aminotransferase
AST: aspartate transaminase
AFP: alpha-fetoprotein
PLT: platelet
TBIL: total bilirubin
ALB: albumin
OS: overall survival
RFS: recurrence-free survival
HR: hazard ratio
CI: confidence interval
